# Increased radiation from Chernobyl decreases the expression of red colouration in natural populations of bank voles (*Myodes glareolus*)

**DOI:** 10.1038/srep07141

**Published:** 2014-11-21

**Authors:** Zbyszek Boratyński, Philipp Lehmann, Tapio Mappes, Timothy A. Mousseau, Anders Pape Møller

**Affiliations:** 1 CIBIO/InBIO, Research Center in Biodiversity and Genetic Resources, University of Porto, PT-4485-661 Vairão, Portugal; 2 Centre of Excellence in Biological Interactions Research, Department of Biological and Environmental Science, P.O. Box 35, FI-40014 University of Jyväskylä, Finland; 3 Department of Zoology, SE-106 91, University of Stockholm, Sweden; 4 Department of Biological Sciences, University of South Carolina, Columbia, SC 29208, USA; 5 Laboratoire d'Ecologie, Systématique et Evolution, CNRS UMR 8079, Université Paris-Sud, Bâtiment 362, F-91405 Orsay Cedex, France

## Abstract

Pheomelanin is a pink to red version of melanin pigment deposited in skin and hair. Due to its bright colour, pheomelanin plays a crucial function in signalling, in particular sexual signalling. However, production of pheomelanin, as opposed to its dark alternative, eumelanin, bears costs in terms of consumption of antioxidants important for protection of DNA against naturally produced reactive oxidative species. Therefore, decreased expression of pheomelanin is expected in organisms exposed to severe oxidative stress such as that caused by exposure to chronic ionizing radiation. We tested if variable exposure to radiation among natural populations of bank voles *Myodes glareolus* in Chernobyl affected expression of red colouration in their dorsal fur. The relative redness of dorsal fur was positively correlated with weight, but also negatively correlated with the level of background radiation. These results suggest that the development of the natural red colouration in adult bank voles is affected by ionizing background radiation, and potentially causing elevated levels of oxidative stress. Reduced production of pheomelanin allows more antioxidants to mitigate the oxidative stress caused by radiation. However, changing natural animal colouration for physiological reasons can have ecological costs, if e.g. it causes mismatch with habitat colouration and conspicuousness for predators.

Colouration in animals may be adaptive and have variable functions, from concealment and communication to regulation of physiological processes^1^. Experimental selection on colouration has been demonstrated^2^,^3^, and it has been argued that it can cause rapid evolutionary change^4^,^5^. In mammals colouration mainly depends on deposition of two forms of melanin, red to pink pheomelanin and black to brown eumelanin, during growth of hair. Regulation of expression of two antagonistic genes (Melanocortin-1 receptor and Agouti signalling protein) determines the proportion of expression in the same melanocytes of either the darker or the lighter form^6^. However, production of either of the two melanin forms carry variable costs that can affect expression levels, particularly under stressful condition^7^. Despite of its potential function^8^,^9^ during synthesis of pheomelanin substantial amounts of the most important intracellular antioxidant, glutathione (γ-glutamyl-cysteinyl-glycine), is consumed. Consequently this antioxidant is not available for neutralizing free radicals, accumulation of which can cause oxidative stress, DNA damage and development of diseases^10^. Production of the alternative to pheomelanin, eumelanin, does not carry such a cost, and, therefore, down-regulation of red colour expression may be a useful mechanism for animals exposed to chronic stressors that cause release of free radicals^7^,^11^.

Chronic exposure to ionizing radiation can cause oxidative stress, deterioration of phenotypes, and death, affecting the ecology and evolution of organisms^12^. It has been shown that birds can adapt to chronic and elevated ionizing radiation, where birds exposed to higher doses of radiation have evolved lowered expression of pheomelanin^13^. Such an effect has never been detected in mammals, although they are exposed to higher and more chronic doses of external ionizing radiation than birds as they live closer to or in (e.g. rodents and shrews) media that accumulate radioactive particles, such as soil. To estimate expression level of pheomelanin we measured relative redness of colouration^7^ of dorsal fur in wild rodents, bank voles *Myodes* ( = *Clethrionomys*) *glareolus* (Schreiber, 1780), exposed to variable levels of ionizing radiation in Chernobyl (Fig. 1). We expected to find a negative association between the level of dorsal fur redness and the level of ionizing radiation of soil if the animals' expression of pheomelanin changes either due to adaptation or phenotypic plasticity.

## Results

The relative red colouration of fur was affected by both soil radiation and animal weight (Table 1). Radiation was negatively correlated with relative red colouration of animal dorsal fur (ρ = −0.15, t = −2.05, df = 187, p = 0.042; Fig. 2a), whereas weight was positively correlated with relative red colouration of animal dorsal fur (ρ = 0.30, t = 4.36, df = 187, p = 0.00002; Fig 2b). Weight and radiation were not significantly correlated (ρ = −0.09, t = −1.29, df = 187, p = 0.197). The negative correlation between relative red colouration of dorsal fur and soil radiation was also observed when reflectance spectroscopy was used to assess colour change (Reduced dataset, ρ = −0.59, t = −5.18, df = 49, p = 0.000004; Fig. 3).

The level of radiation of the soil in the locations where animals were trapped varied from 0.01 to 34.69 μSv/h (with median and mean of 0.37 and 2.75). Overall colour luminosity and reflectance of red colour varied from 21 and 28 to 53 and 65 of 8-bit RGB scale (with medians and means of 35.3, 44.6, 35.6 and 45.1, respectively), with relative red colour varying from 0.38 to 0.51 (with median and mean of 0.46). Animal weight varied from 11.9 to 35.1 g (with median and mean of 23.5 and 23.4). Repeatability (coefficient of intraclass correlation^14^) of colour measures estimated from repeated calculations of 21 specimens were significant for all traits (overall luminosity: τ = 0.93, p < 0.001; reflectance of red colour: τ = 0.88, p < 0.001; relative red colour: τ = 0.44, p = 0.0196). Also repeatability of spectroscopy measures of relative fur irradiance, conducted to validate photographic analyses, was significant (τ = 0.51, < 0.001), and spectroscopy measures were significantly correlated with both the reflectance of red (ρ = 0.40, t = 3.01, df = 49, p = 0.004) and the overall luminosity (ρ = 0.34, t = 2.57, df = 49, p = 0.013) calculated from photographs.

The effects of the interaction between soil radiation and animal weight as well as vegetation cover did not have a significant effect [radiation*sex: estimates (± SE) = −3.454e-02 (± 3.157e-02), t = 0.56, p = 0.57; weight*sex: estimates (± SE) = −3.454e-02 (± 3.157e-02), t = −1.09, p = 0.23; vegetation coverage: estimates (± SE) for forest litter = 2.844e-06 (± 4.710e-05), bushes = 1.662e-07 (± 5.833e-05) and canopy = −7.573e-05 (± 4.675e-05) with |t| < 1.7 and p > 0.1] on relative red colouration of animal dorsal fur, and, therefore, they were omitted from the final statistical analyses.

## Discussion

**Figure 1:**
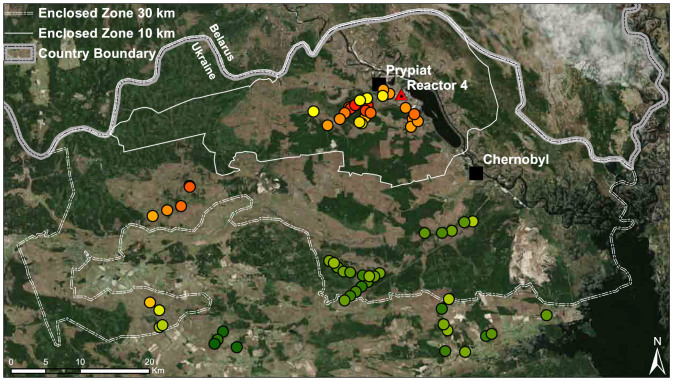
Map of the Chernobyl Exclusion Zone (Ukraine) with locations where bank voles were trapped. The intensity of soil radiation is shown in colour from green (0.01), through yellow to red (34.69 µSv/hour). Figure 1 created using ESRI ArcGIS 10.0. Satellite imagery © CNES/Airbus DS, Earthstar Geographics | Source: Esri, DigitalGlobe, GeoEye, i-cubed, Earthstar Geographics, CNES/Airbus DS, USDA, USGS, AEX, Getmapping, Aerogrid, IGN, IGP, swisstopo, and the GIS User Community | Esri, HERE, DeLorme.

**Figure 2:**
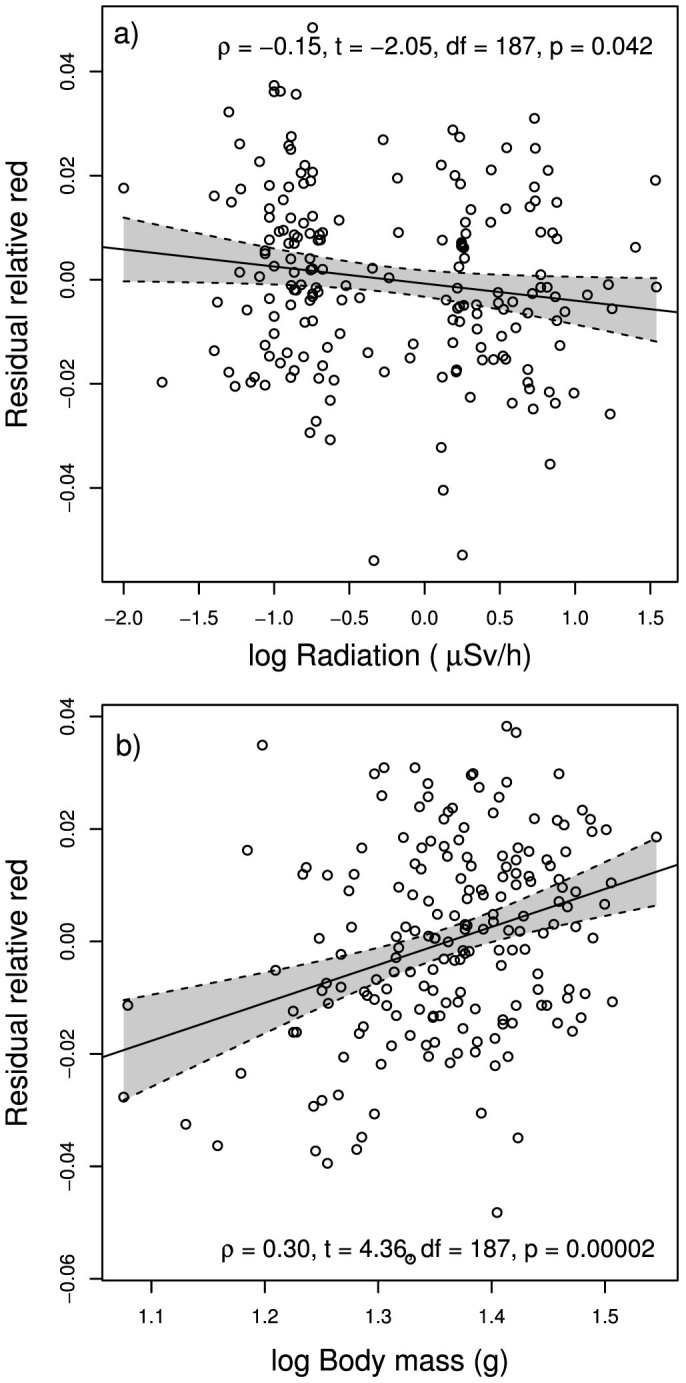
(a) Residual relative red colouration (red/(red+blue+green)) of bank vole dorsal fur regressed against background radiation at ground level (µSv/hour). (b) Residual body mass of bank voles regressed against background radiation at ground level. Residual values of relative red and body mass were calculated from a mixed model including body mass or relative red as predictors and trapping year and location as random factors. Dashed lines (and grey shadings) refer to 95% confidence interval limits of the regression (continuous) lines.

**Figure 3:**
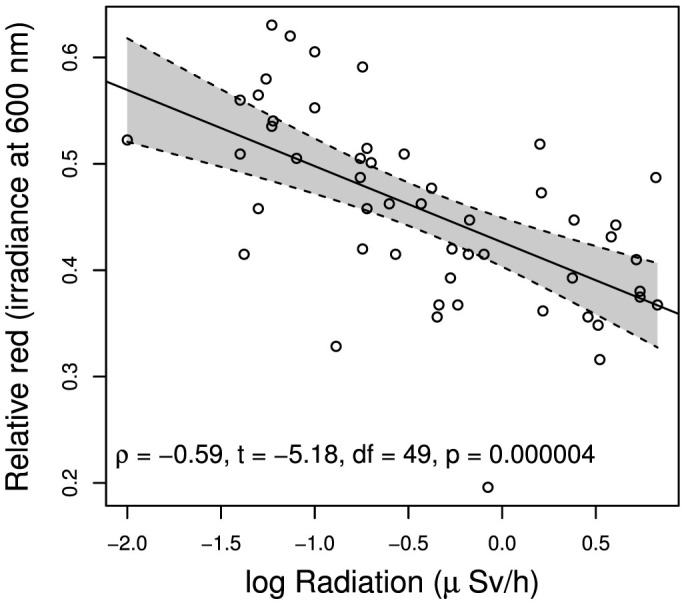
Relative red colouration (relative irradiance of red colour measured at 600 nm) of dorsal skin of bank vole specimens regressed against background radiation at ground level (µSv/hour). Dashed lines (and grey shadings) refer to 95% confidence interval limits of the regression (continuous) lines.

In this study we showed that expression of the relative red colour of dorsal fur in a common rodent depended on the weight of specimens, but also on the level of ionizing radiation of the habitat in Chernobyl where the animals were trapped (Fig. 1). The results based on the photographic method used here (Fig. 2), were validated and corroborated with a spectrophotometric analysis on a smaller sub-sample (Fig. 3).

While dorsal fur colouration might depend on habitat type^14^,^15^ and affect animal fitness^2^,^5^, here we did not find an association between animal colouration and vegetation coverage (i.e. descriptions of habitat type in the forest ecosystem). Rather we found that the relative redness of dorsal fur was a function of animal size, which is a surrogate of age, suggesting that bigger, and older, animals express brighter colouration (Table 1). This is expected as adult colouration in this species is reddish while pups are born greyish^16^. At the same time the effect of animal size and the relative amount of red colour of dorsal fur was significantly explained by radioactive contamination in the trapping area measured here as ionizing radiation (Fig. 2A, Fig. 3). The size of animals and habitat contamination were not correlated with each other. But as the effect of radiation on colouration (Fig. 2a) was opposite in direction to the effect of body size (Fig. 2b), it suggests that ionizing radiation compromises the expression of adults' natural fur colouration and/or there might be selection for larger size in more contaminated areas.

The ecological significance of the decrease of relative red colouration in Chernobyl bank vole populations is speculative as there are no data showing the importance of this character for performance in this species. In other rodent species dorsal colouration is often cryptic and conspicuous animals are more easily found by predators^2^. Nevertheless, it is likely that rodents experiencing conditions of chronic oxidative stress, such as around nuclear disaster sites (e.g. Chernobyl and Fukushima), can down-regulate their expression of pheomelanin, or alternatively, have evolved adaptive responses similar to those observed for birds^13^ whereby low expression levels of pheomelanin, is associated with mitigation of oxidative stress.

## Methods

### Study animals and study area

Bank voles were captured with Ugglan Special 2 live traps (Grahnab, Sweden), with sunflower seeds and potato as bait, during 2011 (June), 2013 (September) and 2014 (June) from 68 locations in the Chernobyl region of Ukraine (Fig. 1). At each location 8–20 traps were placed, with an inter-trap distance of 10 m. Traps were set for two nights. In 2011, 2013, and 2014, 51 (17 females, 34 males), 11 (5 females, 6 males) and 127 voles (53 females, 74 males) were captured, respectively. Vegetation cover (%) was estimated within 1 m radius from each trap. The vegetation cover was estimated in three layers: forest litter (0–50 cm), bushes (0.5–2 m) and canopy (above 2 m). Animals captured in 2011 and 2013 were sacrificed by cervical dislocation and stored at −20°C. Animals captured in 2014 were released back to their original trapping locations. All animals were weighed (Mettler Toledo XS204, to the nearest 0.1 g). All procedures were performed in accordance with relevant guidelines and regulations. The study was approved by the Finnish Ethical Committee (license numbers: ESLH-2008-04660/Ym-23 and ESLH-2009-09663/Ym-23).

### Measurements of background radiation

Hand-held dosimeters (Inspector, SE International, Inc, Summertown, TN, USA) calibrated to measure Sieverts (Sv) were used to measure soil radiation at the exact trapping sites of each animal. Radiation at capture sites varied from 0.01 to 133.00 µSv/hour, although no animals were trapped in sites with radiation levels above 34.69 µSv/hour.

### Colour analyses

Red coloration is an indicator of the proportion of actual pheomelanin concentration in vertebrates, and an association between visual determinations of reddish colour expression and pheomelanin content have been reported previously^7^. Dorsal fur colour was estimated using digital photography of live animals (2014) or dorsal skin specimens (2011 and 2013) with a Canon EOS 400D digital camera, 18–55 mm (set to 55 mm) 1:3.5–56 lens, and Polaroid 48 macro led ring light source (set on full light with white diffuser), accompanied with Opteca colour and white balance grey card. The original large RAW photographs were white balanced with 250 pixels of white standard in GIMP 2.6.12 (www.gimp.org) and saved in high resolution (3900 × 2600 pixels) TIFF format. Colouration was estimated from a square-shaped area (300 × 300 pixels) of a dorsal part of the skin in Hyper-Utility 2 (www.fujifilm.com). Reflectance of red, blue and green were measured as Red-Green-Blue (RGB, 8-bit) standard values (from 0 to 255) from which relative red colour (red/(red+blue+green) of the dorsal fur was estimated^14^. In order to estimate repeatability of colour measurements, we calculated the intraclass correlation coefficient (τ) for 21 individual samples that were reanalysed without prior knowledge of identity or colouration during the second measurements.

To validate the above method, reflectance spectroscopy was used to measure relative irradiance of red colour in fur of dorsal skin specimens. Light was provided by an Ocean Optics PX-2 light-source (Ocean Optics, Dunedin, FL, USA). Relative irradiance was measured with a MayaPro 2000 spectrometer (Spectrecology, Jasper, GA, USA) and analysed with the SpectraSuite software (Ocean Optics, Dunedin, FL, USA). Prior to measurements and after each tenth measurement the setup was re-calibrated against a Labsphere SRS-99-010 reference surface (Labsphere, North Sutton, NH, USA). The light source and sensor were aligned at an angle to measure irradiance at a distance of 10 cm from the fur. Relative irradiance was measured at 600 nm with a 200 ms integration time. From each dorsal skin sample three measures were taken along the medial line. The average of these measures was used for analyses. Furthermore, 19 animals were re-analysed on three consecutive days to estimate repeatability.

### Statistical analyses

As the distribution of soil radiation was right skewed all analyses were performed on log_10_ transformed values of continuous variables. Statistical significance of effects explaining variation in relative redness [mean luminosity of red/(mean luminosity of red + mean luminosity of green + mean luminosity of blue)] of dorsal fur was tested with models that included soil radiation, animal weight (continuous predictors), sex (fixed effect), interactions between continuous predictors and sex, and random effects of population of origin (68 levels) and year (3 levels). Vegetation cover and interactions with sex were omitted from the final model as they were not significant. R Project packages “lm4”, for generalized linear mixed model, and “LMERConvenienceFunctions”, for log likelihood ratio test were used.

## Figures and Tables

**Table 1:** Results of mixed model testing the effects of soil radiation, body mass and sex on the relative redness of dorsal fur of bank voles from Chernobyl

Effect	Estimate	SE	t	P
Intercept	−0.4390	0.0236	−18.60	
Sex	−0.0012	0.0026	−0.475	0.627
Soil radiation	−0.0034	0.0016	−2.071	0.040
Body mass	0.0687	0.0156	4.410	0.00001

Three parameters and their factorial interactions were tested using restricted maximum-likelihood procedures. Sampling year and location were included as random factors, with variance ± SD of 2.34E-004 ± 1.53E-002 and 8.748E-019 ± 9.35E-010, respectively. Number of observations - 189, number of groups: year - 3, locations - 68.

## References

[b1] Caro T. The adaptive significance of coloration in mammals. Bioscience 55, 125 (2005).

[b2] Vignieri S. N., Larson J. G. & Hoekstra H. E. The selective advantage of crypsis in mice. Evolution 64, 2153–8 (2010). 20163447 10.1111/j.1558-5646.2010.00976.x

[b3] Kaufman D. W. Adaptive coloration in *Peromyscus polionotus*: experimental selection by owls. J. Mammal. 55, 271–283 (1974).

[b4] Linnen C. R. *et al.* Adaptive evolution of multiple traits through multiple mutations at a single gene. Science 339, 1312–6 (2013). 23493712 10.1126/science.1233213 PMC3836219

[b5] Karell P., Ahola K., Karstinen T., Valkama J. & Brommer J. E. Climate change drives microevolution in a wild bird. Nat. Commun. 2, 208 10.1038/ncomms1213 (2011). 21343926 PMC3105316

[b6] Hubbard J. K., Uy J. A. C., Hauber M. E., Hoekstra H. E. & Safran R. J. Vertebrate pigmentation: from underlying genes to adaptive function. Trends Genet. 26, 231–9 (2010). 20381892 10.1016/j.tig.2010.02.002

[b7] Galván I., Erritzøe J., Wakamatsu K. & Møller A. P. High prevalence of cataracts in birds with pheomelanin-based colouration. Comp. Biochem. Physiol. A. Mol. Integr. Physiol. 162, 259–64 (2012). 22487483 10.1016/j.cbpa.2012.03.012

[b8] Galván I., Ghanem G. & Møller A. P. Has removal of excess cysteine led to the evolution of pheomelanin? Pheomelanogenesis as an excretory mechanism for cysteine. Bioessays 34, 565–8 (2012). 22442057 10.1002/bies.201200017

[b9] Galván I. & Møller A. P. Pheomelanin-based plumage coloration predicts survival rates in birds. Physiol. Biochem. Zool. 86, 184–92 (2013). 23434778 10.1086/668871

[b10] Wu G., Fang Y., Yang S., Lupton J. R. & Turner N. D. Recent advances in nutritional sciences glutathione metabolism and its implications for health. J. Nutr. 134, 489–492 (2004). 14988435 10.1093/jn/134.3.489

[b11] Galván I., Mousseau T. A. & Møller A. P. Bird population declines due to radiation exposure at Chernobyl are stronger in species with pheomelanin-based coloration. Oecologia 165, 827–35 (2011). 21136083 10.1007/s00442-010-1860-5

[b12] Metcalfe N. B. & Alonso-Alvarez C. Oxidative stress as a life-history constraint: the role of reactive oxygen species in shaping phenotypes from conception to death. Funct. Ecol. 24, 984–996 (2010).

[b13] Galván I. *et al.* Chronic exposure to low-dose radiation at Chernobyl favours adaptation to oxidative stress in birds. Funct. Ecol. Early View (2014). 10.1111/1365-2435.12283

[b14] Boratyński Z. *et al.* Large spatial scale of the phenotype-environment colorcolour matching in two cryptic species of African desert jerboas (Dipodidae: *Jaculus*). PLoS One 9, e94342 (2014). 24714509 10.1371/journal.pone.0094342 PMC3979769

[b15] Hoekstra H. E. Genetics, development and evolution of adaptive pigmentation in vertebrates. Heredity (Edinb). 97, 222–34 (2006). 16823403 10.1038/sj.hdy.6800861

[b16] Aulagnier S., Haffner P., Mitchell-Jones A. J., Moutou F. & Zima J. [Bank vole *Clethrionomys glareolus*]. Mammals of Europe, North Africa and the Middle East. [Bailey, J. (ed.)] [194] (A&C Black Publishers, London, 2009).

